# Intravoxel incoherent motion imaging used to assess tumor microvascular changes after transarterial chemoembolization in a rabbit VX2 liver tumor model

**DOI:** 10.3389/fonc.2023.1114406

**Published:** 2023-02-28

**Authors:** Zhimei Cheng, Huanrong Qin, Wei Cao, Huizhou He, Shuling Zhang, Yushi Yang, Zhenmin Wang, Xun Zou, Lizhou Wang, Xueqing Huang, Shi Zhou, Shuai Zhang

**Affiliations:** ^1^ Department of Interventional Radiology, the Affiliated Hospital of Guizhou Medical University, China Branch of National Clinical Research Center for Interventional Medicine, Guiyang, China; ^2^ Department of Interventional Radiology, The Affiliated Cancer Hospital of Guizhou Medical University, Guiyang, China; ^3^ The Affiliated Hospital of Guizhou Medical University & Key Laboratory of Environmental Pollution Monitoring and Disease Control, Ministry of Education, School of Public Health, Guizhou Medical University, Guiyang, China; ^4^ Department of Pathology, The Affiliated Hospital of Guizhou Medical University, Guiyang, Guizhou, China; ^5^ Department of Radiology, The Affiliated Hospital of Guizhou Medical University, Guiyang, Guizhou, China

**Keywords:** rabbit VX2 liver tumor, transarterial chemoembolization, microvessel density, intravoxel incoherent motion imaging, glycolytic flux

## Abstract

**Purpose:**

To evaluate the correlation between microvascular density (MVD) and intravoxel incoherent motion (IVIM) magnetic resonance imaging (MRI) parameters and the effect of glycolytic flux after transarterial chemoembolization (TACE) in a rabbit VX2 liver tumor.

**Materials and methods:**

VX2 liver tumor allografts in 15 New Zealand white rabbits were treated with sterile saline (control group, n = 5) or lipiodol-doxorubicin emulsion (experimental group, n = 10). MRI was performed 2 weeks after the procedure to evaluate IVIM parameters, including apparent diffusion coefficient (ADC), pure diffusion coefficient (D), pseudodiffusion coefficient (D*), and perfusion fraction (PF). All animal samples were taken of the tumor and surrounding liver. Immunostaining for CD31, CD34, CD105, and VEGF was used to evaluate MVD. The protein expression of Glut4, HK2, PKM2, LDHA, and MCT1 was determined using western blotting. Pearson correlation tests were used to analyze the relationship between MVD and IVIM parameters.

**Results:**

D* value in the peritumoral region was negatively correlated with CD34 (r = –0.71, *P* = 0.01). PF value positively correlated with CD34 (r = 0.68, *P* = 0.015), CD105 (r = 0.76, *P* = 0.004) and VEGF (r = 0.72, *P* = 0.008) in the peritumoral region. Glut4, HK2, PKM2, and MCT1 in the peritumoral regions were higher in the experimental group than in the control group (all *P* < 0.05).

**Conclusion:**

IVIM parameters were correlated with MVD in the intratumoral and peritumoral regions after TACE in a rabbit liver tumor model. The angiogenesis reflected by MVD may be related to changes of glycolytic flux.

## Introduction

Transarterial chemoembolization (TACE) is often used for the treatment of unresectable hepatocellular carcinoma (HCC) (1). Although the short-term effect of this treatment has been demonstrated in multiple studies, its long-term efficacy is poor, with a 5-year survival rate of less than 30% (2, 3). This limited efficacy over the long term is mainly due to a high rate of tumor recurrence and intrahepatic and extrahepatic metastasis (4, 5). Among factors affecting the efficacy of TACE, tumor microenvironment (TME) changes are of particular importance. For example, hypoxia is a key condition affecting the TME of HCC, and research has shown that the modulation of hypoxia inducible factor-1α can reduce sorafenib resistance and improve the prognosis of patients treated with TACE (6). Another study has similarly shown that elevated pyruvate kinase muscle isozyme M2 (PKM2) expression is associated with treatment resistance and reduced survival in patients receiving TACE (7). A recent meta-analysis demonstrated that higher expression levels of glycolysis markers in tumor tissues are correlated with poorer overall survival (8).

These changes in TME result in the formation of new tumor microvessels, eventually leading to tumor recurrence and metastasis (9). Tumor microvessel density (MVD) is a marker that can be used to evaluate tumor angiogenesis. For instance, cluster designation 34 (CD34) staining has been used to evaluate the growth of neovascularization in HepG2 xenograft tumor-bearing mice (10), and CD31 staining can be used to identify the stages of HCC (11). Other research has shown that CD105 staining is useful in quantifying the formation of new microvessels in HCC (12). It is essential to accurately identify these MVD changes after TACE treatment for HCC.

Magnetic resonance imaging (MRI) is a noninvasive method that can be used to assess various tumor characteristics. More specifically, intravoxel incoherent motion (IVIM) imaging is of particular use. In 1986, researchers proposed using a bi-exponential model to separate the diffusion of water molecules and the perfusion of microcirculation (13). With this method, researchers could obtain the apparent diffusion coefficient (ADC), the pure diffusion coefficient (D), the pseudodiffusion coefficient (D*), and the perfusion fraction (PF), parameters that not only reflect tissue diffusion more accurately but also provide information about tissue microcirculation perfusion (13). However, studies assessing IVIM imaging have differed in their conclusions regarding the role of these parameters in predicting treatment efficacy and assessing tumor MVD. For example, one study found that PF value is associated with MVD in ovarian epithelial tumors (14), and another study found that both PF value and D* value are significantly and positively correlated with CD31 and VEGF staining in A549 tumor-bearing mice (15). Other research has shown that PF values are positively correlated with tumor MVD but that ADC and D* values are not correlated with tumor MVD after treatment with transcatheter arterial embolization and apatinib (16).

In this study, we therefore sought to further evaluate the correlation between IVIM parameters and histopathological MVD after TACE treatment in a rabbit VX2 liver tumor allograft model. Through this research, we hoped to determine the potential value of IVIM imaging in evaluating the therapeutic effect of TACE treatment.

## Materials and methods

### Animals

Male New Zealand white rabbits (aged 8 weeks, weighing 2.0-2.5 kg) were purchased from the Animal Center of Guizhou Medical University (license: SYXK 2018­0001). Of these rabbits, a total of 16 rabbits with similar growth status were selected for this study. All experiments in animal models were conducted following the experimental program approved by the Animal Ethics Committee of Guizhou Medical University and following institutional norms (ethics number: 1900932). All animals were maintained in laminar flow rooms at constant temperature and humidity, with free access to food and water.

### Implantation of VX2 liver tumor

**Figure 1:**
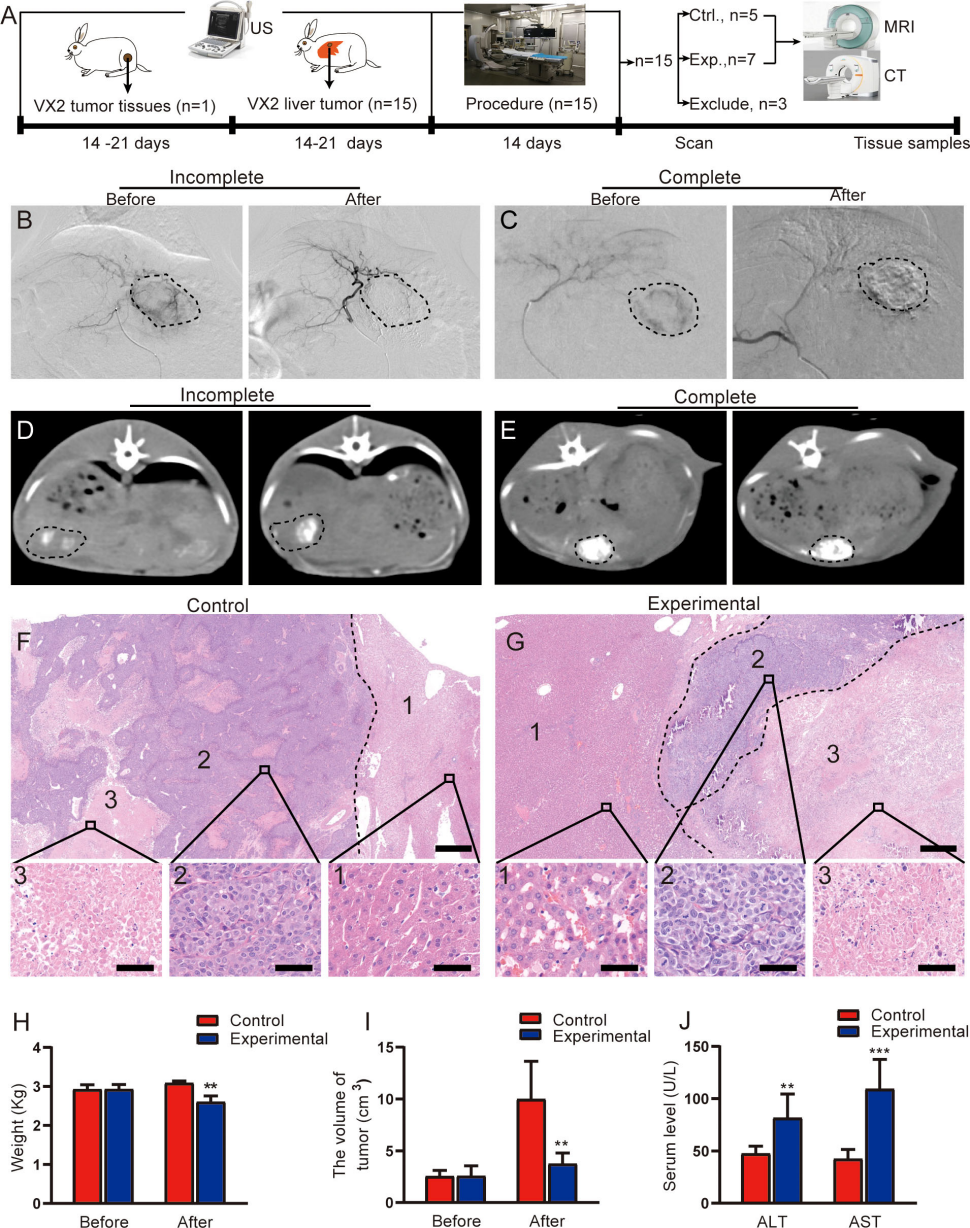
Evaluation of TACE treatment in rabbit VX2 liver tumor models. **(A)** Overview of the experimental design. **(B, C)** Representative angiographic images show the tumor staining before and after embolization in the experimental group. **(D, E)** Representative CT images in the experimental group obtained 2 weeks after TACE. **(D)** shows incomplete lipiodol deposition within the tumor, whereas **(E)** shows complete lipiodol deposition within the tumor. **(F, G)** Representative H&E staining of resected tumor lesions after procedure in the control and experimental groups, consisting of the liver (1), the viable tumor (2), and the necrotic tumor (3). Scale for large image, 500 μm; scale for small image, 50 μm. **(H)** Quantitative analysis of rabbit weight before and after procedure in two groups. **(I)** Quantitative analysis of tumor volume before and after procedure in two groups. **(J)** Quantitative analysis of serum ALT and AST levels after procedure in two groups. Data represent mean ± SD. **P < 0.01; ***P < 0.001. Ctrl., control; Exp., experimental; MRI, magnetic resonance imaging; CT, computed tomography.

VX2 tumor tissues purchased from Guangzhou Gineo Biotechnology Co. (Guangzhou, Guangdong, China) were implanted into the muscle layer of the hindlimb of one carrier rabbit (n = 1) and were grown for 2 to 3 weeks as described in previous study (17). The tumor tissues harvested from the carrier rabbit were then divided and subsequently implanted into the left liver lobe of the remaining rabbits (n = 15) under ultrasound guidance (M6Vet, Shenzhen Mindray Bio-Medical Electronics Co., Guangdong, China) *via* percutaneous puncture. The growth of VX2 tumors was monitored by ultrasonography (**Figure 1A**).

### Experimental and control procedures

All animals underwent either the control or experimental procedure 2 to 3 weeks after tumor implantation, once a well-delineated solitary tumor (1.5-2.0 cm in diameter) could be detected on ultrasound images (18, 19). For these procedures, anesthesia was administered at a concentration of 2.5% to 3.5%, with an oxygen flow rate of 1 L/min. Each rabbit was placed on an operating table containing a digital subtraction angiography (DSA) machine (MEGALIX Cat Plus 125/20/40/80, Siemens Healthcare GmbH, Erlangen, Germany). The procedure was then performed under fluoroscopic guidance as described in a previous study (19). Briefly, the right femoral artery was selected and punctured with a 20G blood vessel puncture needle. A steerable guidewire (M001508110, FATHOMTM-14, Boston Scientific, Marlborough, MA, USA) and microcatheter (105­5091­150, Micro Therapeutics, Irvine, CA, USA) were advanced through the right femoral artery to the celiac axis. The proper hepatic artery was then selectively catheterized off the common hepatic artery with the aid of a steerable guidewire. A mixture of lipiodol and doxorubicin (up to 1.0 mL; experimental group) or sterile water (control group) was slowly infused under fluoroscopic guidance. Hepatic arteriography was performed to detect staining of the tumor before and after embolization. All rabbits can be weighed before and 2 weeks after procedure.

### Inclusion criteria and study groups

Rabbits were eligible for inclusion if the implanted tumor was located in the liver parenchyma and extensive extrahepatic metastasis was not seen. Animals were excluded if significant tumor staining was still observed after infusion with a mixture of lipiodol and doxorubicin (experimental group).

### MR acquisition and image analysis

All MR examinations were performed with the rabbits under deep sedation. A 3.0T scanner (750 W Discovery, GE Medical Systems, Fayetteville, Wisconsin, USA) with an 8-channel rabbit coil (Wankang Medical Technology Co., Jiangsu, China) was used to scan rabbits in the prone position 2 weeks after treatment, as described previously (19). In brief, an axial T2−weighted HASTE sequence was performed using the following parameters: repetition time/echo time, 4454.0/98.7 ms; field of view, 140 × 140 mm; matrix size, 140 × 140; section thickness, 4 mm; gap, 0 mm; and bandwidth, 41.67 Hz/pixel. IVIM imaging was performed in the transverse plane using an echo−planar imaging sequence with diffusion−gradient encoding in three orthogonal directions; scans were performed with single excitation in a free-breathing state. The b values were 0, 10, 20, 50, 80, 100, 150, 200, 400, 800, and 1000 mm^2^/s. The scan parameters for IVIM imaging were as follows: repetition time/echo time, 3585/66.1 ms; slice thickness/layer interval, 4/0 mm; layer number, 16; field of view, 140 × 140 mm; frequency, 64; phase, 2; bandwidth, 166.7 Hz/pixel; frequency coding direction, R/L; matrix, 64 × 80; and acquisition time, ~1 minute and 30 seconds.

One radiologist with 5 years of experience in abdominal MR imaging evaluated all MR images on a GE Healthcare Advantage Workstation; the radiologist was blinded to the histopathological results. The ADC, D, D*, and PF maps derived from IVIM imaging were extracted after fitting with a bi-exponential model. Parametric values were automatically output by measuring the region of interest (ROI) using incorporated software on a commercial workstation (Syngo, Siemens Healthcare). The largest three sections were chosen, and three ROIs measuring 5 to 15 mm^2^ were drawn manually on each section to measure the ADC, D, D*, and PF values of the intratumoral region, peritumoral region, and liver region. The peritumoral region was defined as an area around the tumor of approximately 2 mm (20); the liver region was defined as an area 2 cm away from the tumor margin. The average values of all ROIs were used for statistical analyses. The tumor size was estimated by calculating its largest (L) and smallest (S) diameters using the following formula: Tumor volume/mm^3^ = (L × S^2^)/2 (21).

### CT scan

After MR imaging, computed tomography (CT) imaging was performed to assess lipiodol deposition (22). Each rabbit was sedated and then scanned, in the prone position, on a 128-slice Multislice CT scanner (SOMATOM definition AS+, Siemens Healthcare). The volumetric scanning parameters were as follows: field of view, 22 cm; tube voltage, 120 kVp; tube current, 250 Ma; and slice thickness, 1.0 mm, as described in a previous study (23).

### Conventional liver function tests

Once imaging was complete, blood was collected from an artery in each rabbit’s ear. For each sample, the supernatant was collected after centrifugation at 3000 rotations per minute for 15 minutes at 4°. The levels of alanine aminotransferase (ALT) and aspartate aminotransferase (AST) were assessed using an automatic biochemical analyzer (Chemray 240, Shenzhen Life Science & Technology).

### Pathology

Immediately after blood collection, the rabbits were euthanized under deep anesthesia with isoflurane. Tumor and adjacent liver samples were then collected and fixed in a 10% formalin solution. Paraffin blocks were cut and stained with hematoxylin and eosin (H&E) and immunohistochemistry stains for CD31, CD34, CD105, and vascular endothelial growth factor (VEGF). The sections were placed in boiling citrate buffer for 20 minutes for antigen repair. Tissue sections were incubated in endogenous peroxidase reagent (ORIGENE, Beijing, China) for 10 minutes at room temperature to block the endogenous peroxidase of cells, and the samples were then incubated with 10% goat serum for 20 minutes at 37°. Incubation with primary antibodies occurred at 4° in hydration chambers overnight. The anti-mouse CD31, CD34, CD105, and VEGF antibodies were purchased from Aifang Biological Co. (Hunan Province, China).

Image J software (Wayne Rasband National Institutes of Health, USA) was used to quantify CD31, CD34, CD105, and VEGF staining. The percentage of staining in each region was determined by dividing the area of staining by the total area of the region. The percentage of positively stained area was calculated in at least six fields per section. All sections were analyzed and evaluated independently by two double-blinded pathologists, and the results were reconfirmed by a third pathologist once inconsistent.

### Western blotting

RIPA lysate (Solarbia, Beijing, China) was used to extract the total protein from the intratumoral, peritumoral and liver regions respectively. Equal amounts of protein were separated on 10% SDS-PAGE gels and transferred to a PVDF membrane (Amersham Hybond, GE Healthcare). Protein detection was performed using anti-glucose transporter 4 (Glut4, Abcam Cat# ab33780, RRID : AB_2191441), anti-hexokinase 2 (HK2, LSBio (LifeSpan) Cat# LS-B3571-50, RRID : AB_10622121), anti-PKM2 (Novus Cat# NBP1-48308, RRID : AB_10011057), anti-lactate dehydrogenase A antibody (LDHA, Abcam Cat# ab47010, RRID : AB_1952042), anti-monocarborxylate transporter 1 (MCT1, Absin, Shanghai, China, cat# abs120479), anti-β-actin (Enogene, Nanjing, China, cat# E12-041), anti-mouse-horseradish peroxidase (HRP, Absin, Shanghai, China, cat# abs20001ss), anti-rabbit-HRP (Absin, cat# abs20040), and anti-goat-HRP (Bioss, Wuhan, China, cat# bs-0294D-HRP). Protein detection was performed using ECL Western blotting substrate (Affinity Biosciences, KF003, Jiangsu, China). β-actin was used as a loading control. Blots were quantified using Image J, and intensities of the protein of interest were normalized to β-actin.

### Statistical analysis

SPSS software (version 26; IBM, Armonk, NY, USA) was used for data analysis. Descriptive statistics were reported as mean ± standard deviation (SD). Groups were compared using independent sample *t* tests. A Pearson correlation test was used to analyze the correlation between IVIM parameters and MVD. *P* values *<* 0.05 were considered statistically significant.

## Results

### TACE treatment in rabbit VX2 liver tumor model

The process of tumor implantation and subsequent tumor formation was successful in all 15 rabbits. No periprocedural complications occurred in any of the study animals. Under DSA fluoroscopy, 3 rabbits in the experimental group were found to still have tumor staining after embolization in the experimental group (**Figure 1B**); these animals were therefore excluded from the analysis. The remaining 7 rabbits in the experimental group had no obvious tumor staining (**Figure 1C**). Of these 7 rabbits, 3 demonstrated incomplete lipiodol deposition in tumors on CT imaging (**Figure 1D**), and the remaining 4 demonstrated relatively complete lipiodol deposition (**Figure 1E**).

In the control group (n = 5), H&E staining demonstrated a large number of viable tumor cells in the intratumoral region, with disordered arrangement, large cell volume, and imbalanced ratio of nucleus to cytoplasm small irregular necrotic areas were also seen in the viable tumor region (**Figure 1F**). In the experimental group, H&E staining demonstrated complete tumor necrosis in 2 of the 7 rabbits; staining in the remaining 5 rabbits demonstrated a few viable tumor cells in the necrotic tumor rim (**Figure 1G**). The liver lobular architecture in the control group was well preserved, and no edema, congestion, or centrilobular necrosis was observed. The experimental group displayed focal hepatocyte necrosis and infiltration of inflammatory cells, as well as sinusoidal congestion.

The body weight of the rabbits was significantly lower in the experimental group than in the control group (t = 3.98, *P* = 0.003; **Figure 1H**). This may be related to little or no food consumption in rabbits after TACE. The tumor volume in the experimental group was also significantly lower than the volume in the control group (t = 3.724, *P* = 0.017; **Figure 1I**. TACE treatment inhibited tumor growth.

Postoperative changes in serum ALT and AST levels are shown in **Figure 1J**. Serum ALT and AST significantly higher in the experimental group than in the control group (ALT: t = 3.708, *P* = 0.007; AST: t = 5.863, *P* < 0.001).

### Quantitative analysis of tumor and liver using MR IVIM parameters

**Figure 2:**
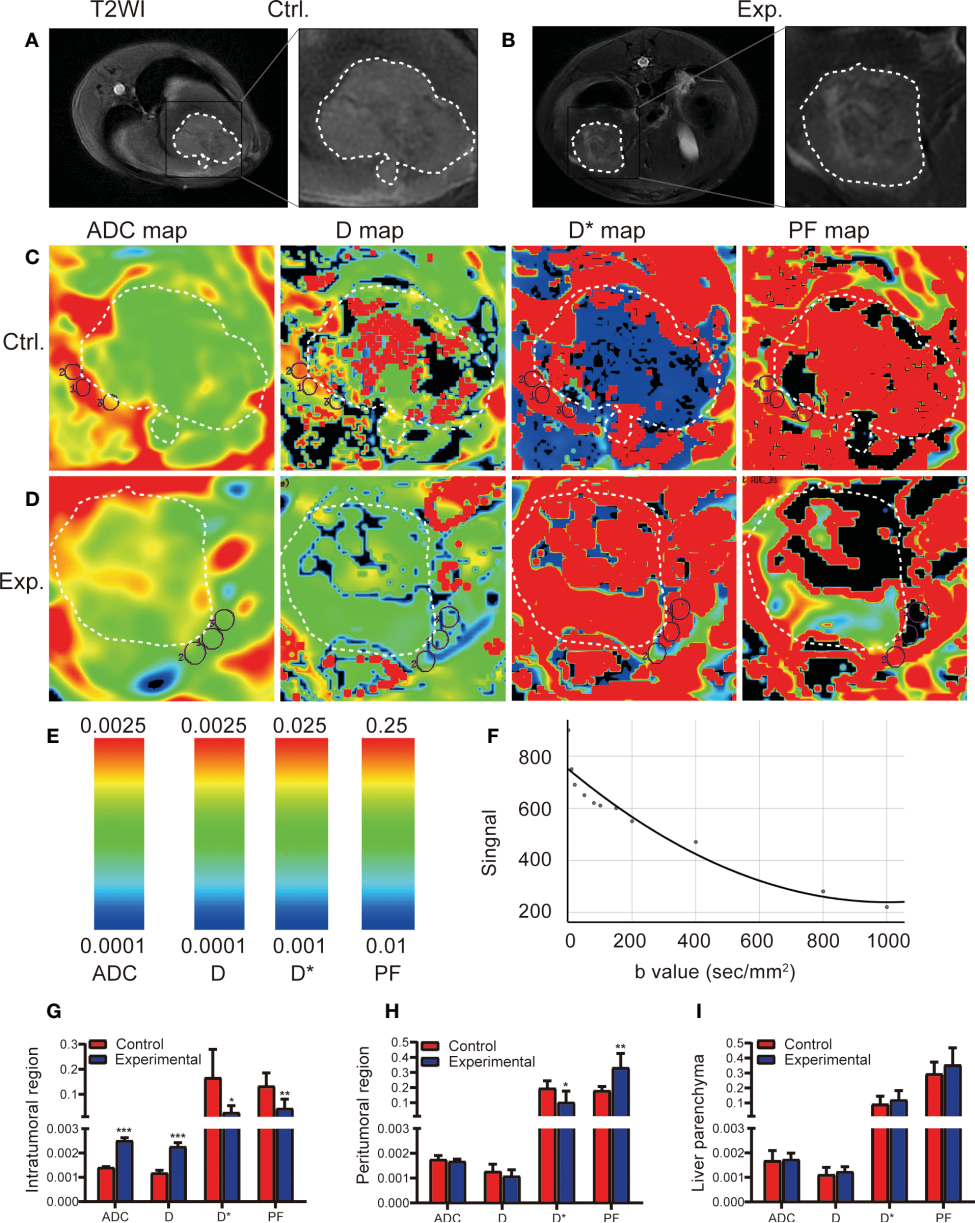
IVIM MRI was used to scan the rabbit VX2 liver tumor model 2 weeks after the control or experimental procedure. **(A, B)** Representative axial T2-weighted images (T2WI) of the tumor in the control and experimental groups delineate the tumor area 2 weeks after the procedure. **(C, D)** ADC, D, D*, and PF maps derived from the IVIM sequence for the control and experimental groups. **(E)** Scale of IVIM parameter value. **(F)** IVIM fitting curve. **(G-I)** Quantitative analysis of IVIM parameters for the intratumoral, region, peritumoral region, and liver parenchyma in the control and experimental groups. Data represent mean ± SD. *P < 0.05; **P < 0.01; ***P < 0.001. Ctrl., control; Exp., experimental; ADC, apparent diffusion coefficient; D, pure diffusion coefficient; D*, pseudodiffusion coefficient; PF, perfusion fraction.

On T2-weighted imaging, central necrosis demonstrated low T2 signal intensity, whereas viable tumor tissue demonstrated slight T2 hyperintensity (**Figures 2A, B**). In the experimental group, an uneven signal intensity was seen in the intratumoral region (**Figure 2B**). IVIM sequence color maps are shown in **Figures 2C, D**, and the scale of the color maps and the fitting curves with IVIM sequences are shown in **Figures 2E, F**. Compared with the control group, the intratumoral ADC and D values in the experimental group were significantly increased (t = 23.256, *P* < 0.001; t = 13.53, *P* < 0.001, respectively), and the D* and PF values were significantly decreased (t = 23.256, *P* < 0.001; t = 13.53, *P* < 0.001, respectively) (**Figure 2G**), which means that the expansion of water molecules in the intratumoral region increases, and the blood flow and velocity in the capillaries decrease after TACE. Compared with the control group, the D* value in the peritumoral region was significantly decreased in the experimental group (t = 2.478, *P* = 0.033), and the PF value was significantly increased (t = 4.069, *P* = 0.004) (**Figure 2H**), which means that may be related to the increase of peritumoral new microvessels. The ADC, D, D*, and PF values in the liver parenchyma were similar between the two groups (**Figure 2I**).

### The correlation between MVD and IVIM parameters after treatment

**Figure 3:**
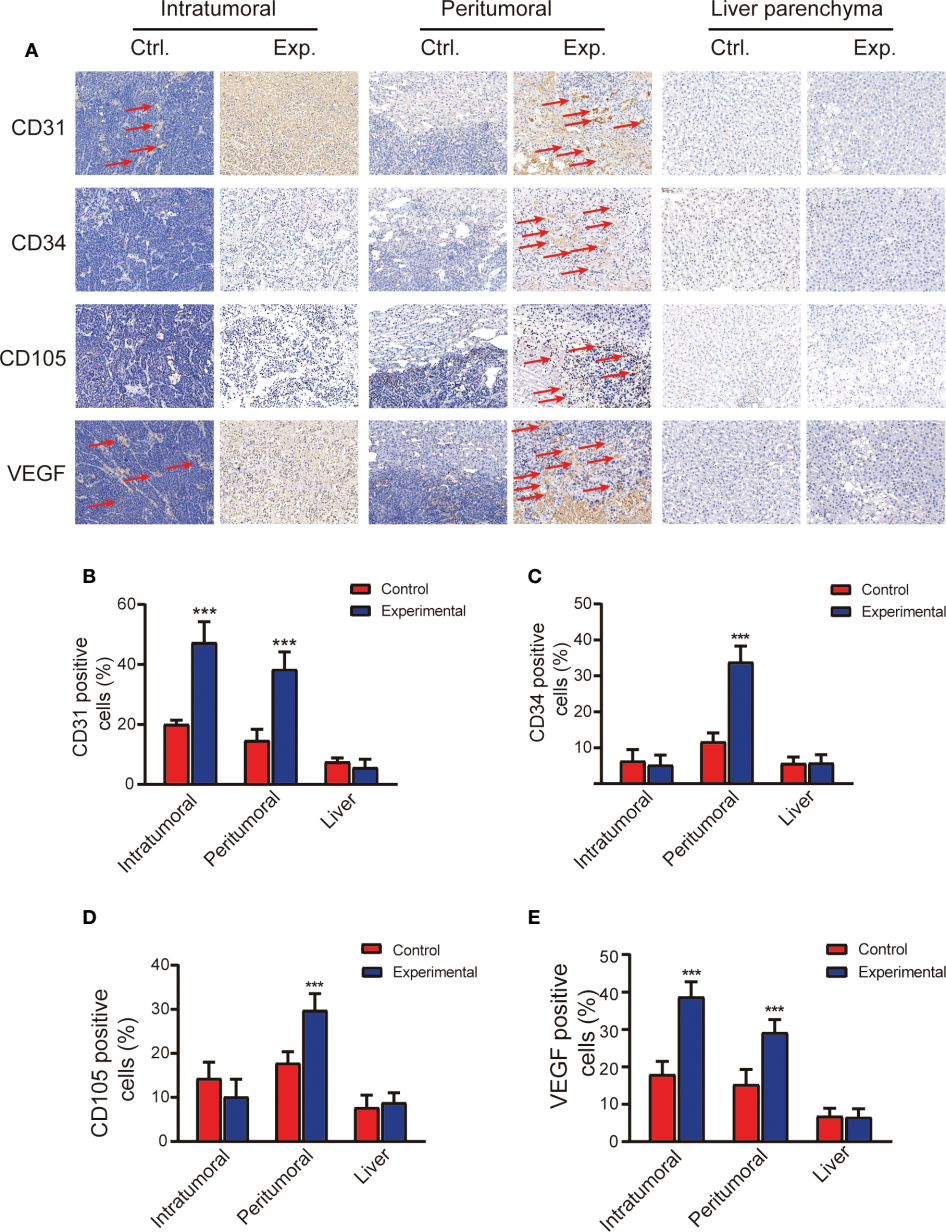
Immunohistopathology staining demonstrating microvasculature in the tumor and liver 2 weeks after the control or experimental procedure. **(A)** Representative images of the intratumoral, peritumoral, and liver regions stained with anti-CD31, anti-CD34, anti-CD105, and anti-VEGF. Scale bars, 100 μm. **(B-E)** Quantitative analysis of CD31, CD34, CD105, and VEGF staining. Data represent mean ± SD. ***P < 0.001. Ctrl, control; Exp., experimental; VEGF, vascular endothelial growth factor.

CD31, CD34, CD105, and VEGF staining results, which are widely used to characterize MVD, are shown in **Figure 3A**. The percentage of CD31 and VEGF staining in the intratumoral and peritumoral regions was significantly higher in the experimental group than in the control group (t = 8.607, *P* < 0.001; t = 7.992, *P* < 0.001, respectively); there were no differences in the liver parenchyma between the two groups. The percentage of CD34 and CD105 staining in the peritumoral region was significantly higher in the experimental group than in the control group (t = 10.101, *P* < 0.001); there were no significant differences between the two groups in the intratumoral region or in the liver parenchyma (**Figures 3B-E**).

**Figure 4:**
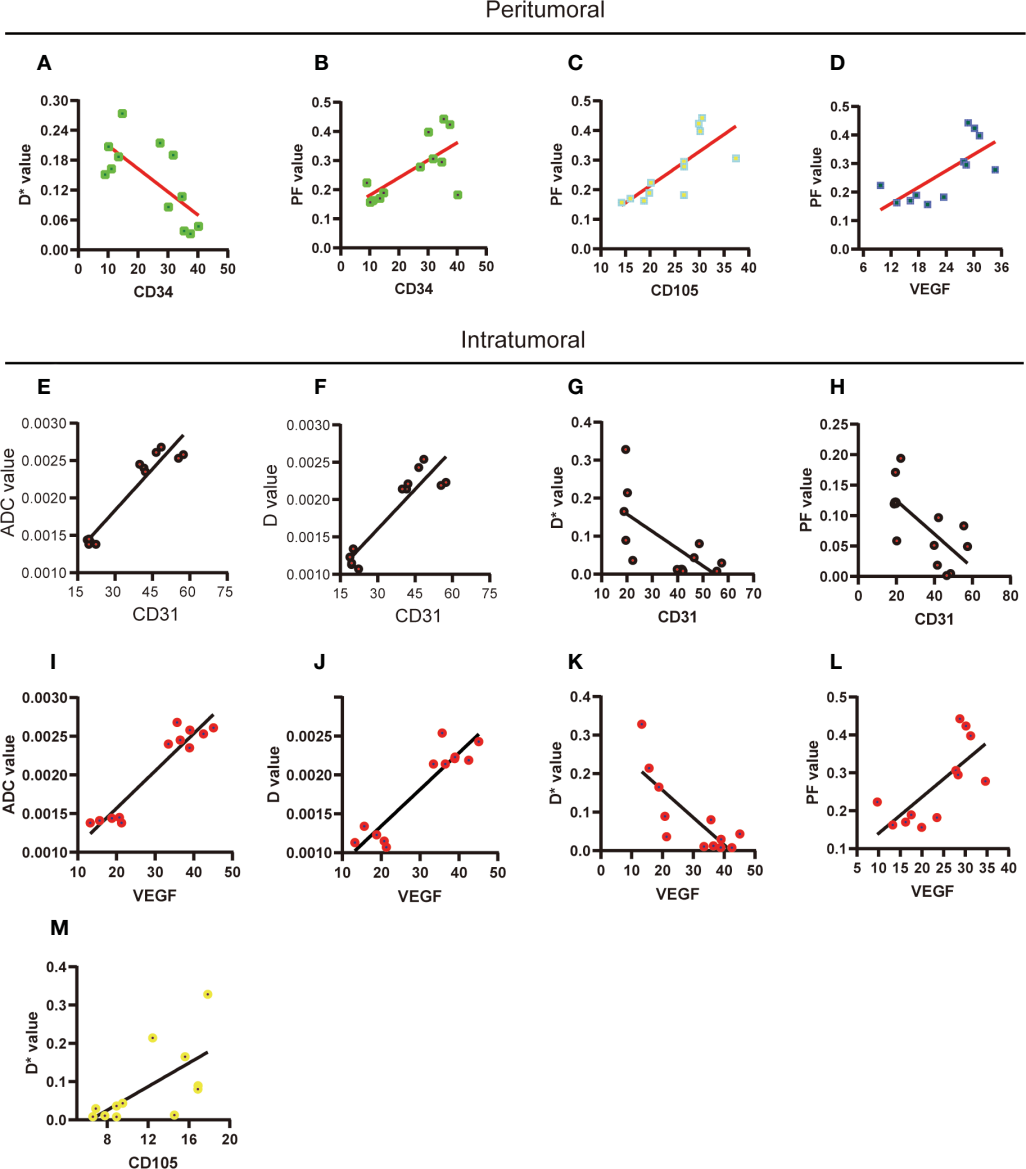
Correlations between the quantitative results of histological staining and IVIM parameters. **(A-D)** The significant correlation between histological staining and IVIM parameters in peritumoral region was performed. **(E-M)** The significant correlation between histological staining and IVIM parameters in intratumoral region was performed. ADC, apparent diffusion coefficient; D, pure diffusion coefficient; D*, pseudodiffusion coefficient; PF, perfusion fraction.

Analysis of the correlation between immunohistochemistry staining and IVIM parameters is shown in **Table 1**. In the peritumoral region, the D* value was negatively correlated with CD34 (**Figure 4A**), and the PF value was positively correlated with CD34, CD105, and VEGF (**Figures 4B-D**). In the intratumoral region, the ADC and D values were positively correlated with CD31 and VEGF, and D* and PF values were negatively correlated with CD 31 and VEGF (**Figures 4E–L**) and the D* value was positively correlated with CD105 (**Figure 4M**).

**Table 1:** The correlation between MVD and IVIM parameters.

Region	Parameter	CD31		CD34		CD105		VEGF	
		r	*P*-value	r	*P*-value	r	*P*-value	r	*P*-value
Intratumoral	ADC	0.957	<0.001***	–0.184	0.567	–0.44	0.152	0.950	<0.001***
	D	0.924	<0.001***	–0.0258	0.418	–0.418	0.177	0.927	<0.001***
	D star	–0.673	0.016*	0.294	0.354	0.652	0.022*	–0.795	0.002**
	PF	–0.662	0.019*	0.325	0.303	0.184	0.567	–0.614	0.034*
Peritumoral	ADC	–0.317	0.316	–0.269	0.397	–0.246	0.441	0.283	0.373
	D	–0.214	0.504	–0.255	0.425	–0.418	0.176	–0.265	0.406
	D star	–0.565	0.055	–0.706	0.01*	–0.477	0.117	–0.371	0.236
	PF	0.528	0.078	0.68	0.015*	0.761	0.004**	0.72	0.008**
Liver parenchyma	ADC	0.345	0.272	–0.275	0.387	–0.088	0.786	–0.159	0.622
	D	–0.059	0.855	–0.548	0.065	–0.269	0.398	0.176	0.584
	D star	–0.286	0.367	0.315	0.319	–0.007	0.984	0.490	0.106
	PF	–0.006	0.986	–0.391	0.209	–0.125	0.698	–0.547	0.066

MVD, microvessel density; IVIM, intravoxel incoherent motion; CD31, cluster designation 31; CD34, cluster designation 34; CD105, cluster designation 105; VEGF, vascular endothelial growth factor; ADC, apparent diffusion coefficient; D, pure diffusion coefficient; D star, pseudodiffusion coefficient; PF, perfusion fraction. *P < 0.05; **P < 0.01; ***P < 0.001.

### The protein expression of glycolytic flux was increased after TACE treatment

**Figure 5:**
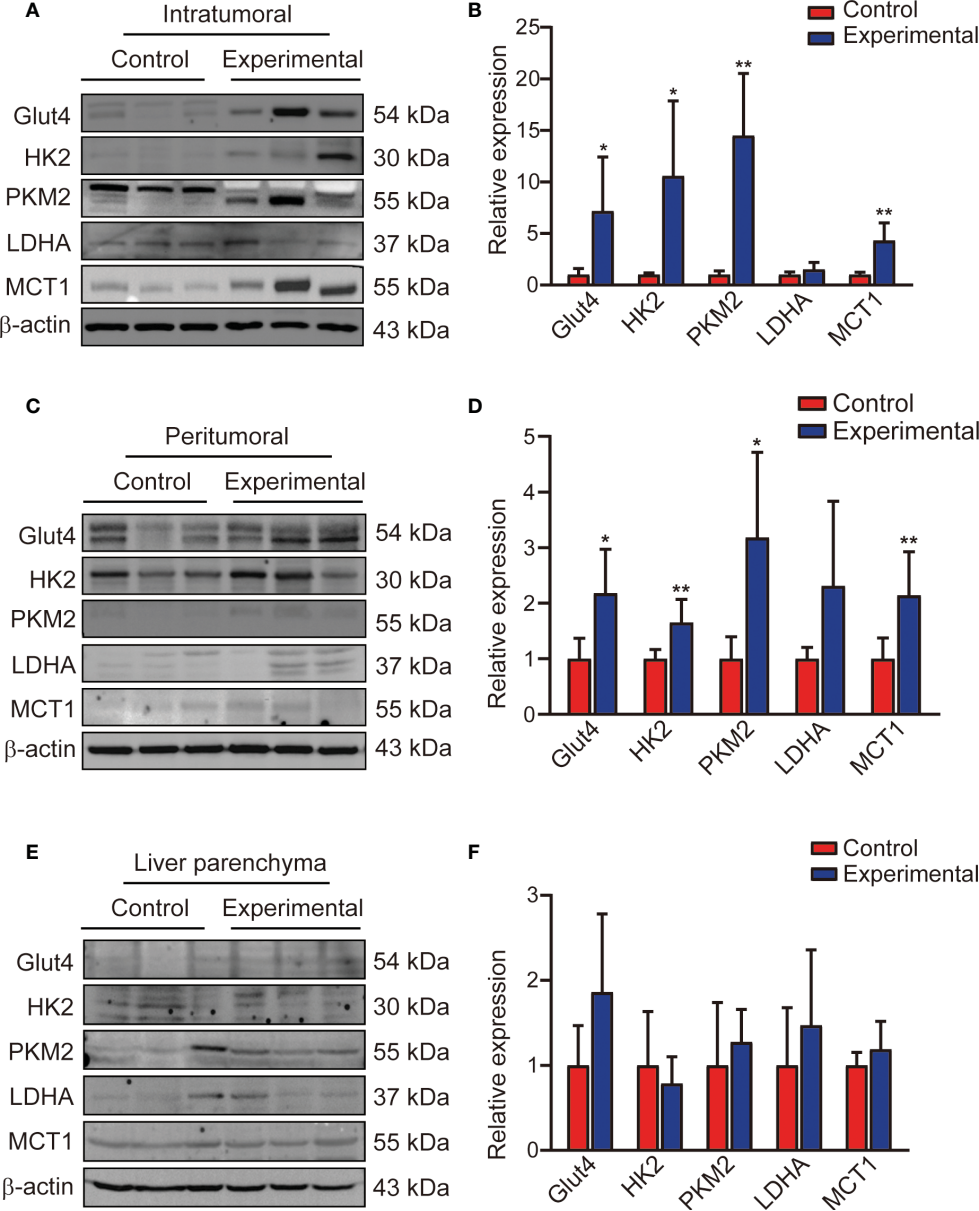
Expression of glycolysis flux protein in the intratumoral region, peritumoral region, and liver parenchyma. **(A, B)** Western blotting and corresponding bar chart for the intratumoral region, **(C, D)** the peritumoral region, **(E, F)** and the liver parenchyma. Data represent mean ± SD. *P< 0.05; **P < 0.01. Glut4, glucose transporter 4; HK2, hexokinase 2; PKM2, pyruvate kinase muscle isozyme M2; LDHA, lactate dehydrogenase A; MCT1, monocarboxylate transporter 1.

As a key enzyme in glycolytic metabolic, the expression levels of Glut4, HK2, PKM2, and MCT1 were significantly higher in the experimental group than in the control group in the intratumoral (t = 2.58, *P* = 0.027; t = 3.47, *P* = 0.013; t = 5.91, *P* = 0.001; t = 4.21, *P* = 0.002, respectively) and peritumoral (t = 3.07, *P* = 0.012; t = 3.77, *P* = 0.005; t = 3.07, *P* = 0.012; t = 3.34, *P* = 0.009, respectively) regions. LDHA expression increased slightly after TACE treatment, but this increase was not significant in the intratumoral region or in the peritumoral region (**Figures 5A-D**). The expression levels of Glut4, HK2, PKM2, LDHA, and MCT1 in the liver parenchyma were similar between the two groups (**Figures 5E, F**).

## Discussion

In this study, we found that the IVIM parameters ADC, D, D*, and PF were associated with MVD changes in the intratumoral and peritumoral regions after TACE treatment in a rabbit VX2 liver tumor model. These parameters, especially PF value, were particularly useful in the evaluation of neovascularization of the peritumoral region. The expression of the protein enzymes of glycolytic flux in the intratumoral and peritumoral regions was also enhanced after TACE treatment.

Previous studies have shown that many factors can negatively affect the long-term efficacy of TACE, including incomplete tumor-supplying arterial embolization, establishment of complex trophic arterial collateral circulation, involvement of the portal vein, residual tumor blood supply, and tumor hypoxia after embolization. All of these factors can lead to tumor angiogenesis (24–26). The degree of angiogenesis can be assessed most directly by using immunohistochemistry staining to evaluate MVD. Previous research in a rabbit VX2 liver tumor model demonstrated that tumor angiogenesis begins to appear in the residual tumor and tumor junction 14 days after chemoembolization with lipiodol (27). In this study, MVD in the intratumoral and peritumoral regions was significantly higher in the experimental group than in the control group, especially in the peritumoral region; these findings are consistent with the results of previous research (27, 28).

In this study, the D values in the liver and tumor were lower than the ADC values in any group in any area, consistent with previous reports (29). This suggests that the ADC value is affected by the diffusion movement of microcirculatory perfusion. Previous research has demonstrated that the diffusion of water molecules is limited by the presence of viable tumor cells, leading to restricted cellular space (30), and that the vascular structure is destroyed and the blood supply is blocked after TACE (31). In this study, we found that the ADC and D values of the intratumoral region in the experimental group were higher than those in the control group, whereas the D* and PF values were lower. The presence of massive necrotic cells in the experimental group after TACE increased water movement and reduced microcirculatory perfusion. Interestingly, the D* value of the peritumoral region was significantly lower in the experimental group than in the control group, whereas the PF value was significantly higher. The D* value mainly characterizes changes in hemodynamics, which depend on the length and flow rate of blood vessels (32). Although the number of new microvessels in the peritumoral area increased, the immature blood vessels still lacked the typical vascular structure and had high vascular permeability, so the blood flow of the microvessels in this area was slow, as has been shown previously (22). The PF value is defined as the fractional volume of capillary blood flowing in each imaging voxel. Our data demonstrated that PF value can be illuminated with abundant new neovascularization in the peritumoral region after TACE.

From an angiogenic standpoint, CD31, CD34, CD105, and VEGF are widely used to characterize MVD in the field of tumor research. Previous research in a VX2 tumor model demonstrated that the expression of CD31 is significantly increased at 20 days after TACE (22). In this study, VEGF and CD31 were strongly expressed in the intratumoral and peritumoral tissues. We found that most of the CD34 and CD105 expressed in the peritumoral tissue represented new tumor vessels after TACE treatment; these factors were barely expressed in the intratumoral region in the experimental group. Similarly, a previous study demonstrated that CD105 was highly correlated with postoperative recurrence and metastasis in HCC (12). Overall, these findings suggest that MVD, especially CD105, is a navel marker for tumor angiogenesis in liver VX2 tumor model after TACE.

Previous studies have shown that PF is associated with CD31 staining of the tumor (14, 33) and that D* and PF values are positively correlated with CD31 and VEGF (15, 34). In the current study, ADC, D, D*, and PF values were positively correlated with CD31 and VEGF in the intratumoral region, and the D* value was also associated with CD105 in the intratumoral region. Other research has demonstrated that D and PF values are positively correlated with CD34 staining after transcatheter arterial embolization combined with apatinib in a VX2 liver tumor model (16). However, in a study of human gastric cancer-bearing nude mice, ADC and D values were found to be associated with tumor necrosis and apoptosis (35). In our study, H&E staining demonstrated a large amount of tumor cell necrosis as a result of TACE treatment. The PF value was also positively correlated with CD34, CD105, and VEGF in the peritumoral region, representing the active and viable area; this finding is consistent with previous results (15, 36). Because the state of microvessels is directly related to tumor growth, invasion, metastasis, and prognosis (37), our study suggests that PF values derived from IVIM imaging may theoretically be useful as a marker of these variables.

Previous studies regarding tumor glycolysis have found that tumor tissues preferentially use glycolytic metabolism and that tumor recurrence and metastasis are associated with enhanced glycolytic metabolism; thus, antiglycolytic key enzymes could potentially play a role in antitumor treatment (7, 38–41). In one study, elevated PKM2 was found to be associated with treatment resistance and shortened survival in patients undergoing TACE treatment, suggesting that PKM2 knockdown could improve TACE efficacy (7). In the current study, TACE was found to induce the expression of glycolytic proteins, including HK2 and PKM2. In addition, the high expression of Glut4 was found to mediate increased glucose uptake, and the high expression of MCT1 was found to induce acidification of the TME. Several studies have shown that acidification of the TME is related to a metabolic shift of cancer cells to a hyperglycolytic phenotype, which is associated with poor survival (42, 43). Therefore, high glycolytic flux may be associated with a poorer prognosis.

This study had several limitations. Performing MRI under free breathing may have affected the accuracy of the results. In addition, a rabbit’s stomach cavity is large, potentially leading to artifacts in images of the liver parenchyma near the stomach. Considering the 3-dimensional structure of the liver and tumor, parameters measured in only part of the transverse position of the tumor likely do not represent the parameters in the entire tumor, and it is difficult to correlate these results with pathologic findings. Finally, tumors in the experimental group had varying degrees of necrosis, complicating the analysis.

In conclusion, this study demonstrated that the IVIM parameters ADC, D, D*, and PF are associated with tumor MVD after TACE in a rabbit VX2 liver tumor model. These results suggest that IVIM parameters may be useful as quantitative biomarkers for the characterization of angiogenesis and that these parameters could potentially be used to evaluate changes in tumor microcirculation after TACE in patients with HCC. In addition, our findings suggest that changes in the protein enzymes of glycolytic flux induced by TACE treatment may be associated with tumor angiogenesis.

## Data availability statement

The original contributions presented in the study are included in the article/supplementary material. Further inquiries can be directed to the corresponding author.

## Ethics statement

The animal study was reviewed and approved by the Animal Ethics Committee of Guizhou Medical University and following institutional norms (ethics number: 1900932).

## Author contributions

ShiZ and ShuaZ contributed to the experiment design, and data analysis. HQ and WC contributed to the experiment implementation, ZC contributed to manuscript draft and data analysis. All authors contributed to the article and approved the submitted version.
